# Visible light and/or UVA offer a strong amplification of the anti-tumor effect of curcumin

**DOI:** 10.1007/s11101-013-9296-2

**Published:** 2013-05-07

**Authors:** August Bernd

**Affiliations:** Clinic for Dermatology, Venereology and Allergology, Johann Wolfgang Goethe University, Theodor-Stern-Kai 7, 60590 Frankfurt, Germany

**Keywords:** Natural anti cancer drugs, Apoptosis, Cell growth arrest, Photodynamic therapy

## Abstract

Curcumin, a dietary pigment from the plant *Curcuma longa*, inhibits cell proliferation and induces apoptosis in different cell lines. The therapeutic benefit is hampered by a very low absorption after trans-dermal or oral application. Therefore, great efforts were undertaken to enhance the effectiveness of curcumin. Recently, it was demonstrated that curcumin offers the described effects also at low concentrations (0.2–1 μg/ml) when applied in combination with UVA or visible light. The efficacy of this combination was shown in human epidermal keratinocytes and in a panel of other cell species in vitro as well as in a xenograft tumor model with A431 tumor cells injected subcutaneously in the flanks of NMRI nude mice in vivo. The treatment of keratinocytes with curcumin and light resulted in the inhibition of cell growth, and in the induction of apoptosis, whereas no toxic cell membrane damage was detectable. The treatment of tumor bearing nude mice with curcumin and visible light resulted in reduced tumor volumes, reduced proliferation rates, and the induction of apoptosis in the tumors. On the molecular level inhibition of extracellular regulated kinases 1/2 and epidermal growth factor receptor was observed which may aid to inhibition of proliferation and induction of apoptosis. This review covers the experiences of the new combination treatment of human tumors.

## Introduction

Curcumin is a pharmacologically active substance isolated from the rhizome of the plant *C. longa Lin.* (*Zingiberaceae* family). The plant is used for thousands of years as a remedy in Asian traditional medicine (Araújo and Leon 2001) showing among others anti-inflammatory and anti-oxidative activities (Xu et al. 1997). Since knowing that curcumin inhibits cell growth and induces apoptosis very great efforts were made to elucidate the anti-cancer activities of curcumin identifying the underlying cell physiological mechanisms. In the meantime it became evident that curcumin modulates survival-associated signal path-ways (Chaudhary and Hruska 2003; Squires et al. 2003; Masamune et al. 2006) causing apoptosis in an intrinsic and/or extrinsic manner depending on the cell types studied (Bush et al. 2001; Kim et al. 2001; Chakraborty et al. 2006; Dujic et al. 2007). The anti-tumor effect of curcumin is documented for a lot of different tumors (e.g. Anand et al. 2008). Considering the low almost not existing toxicity of curcumin the development of new therapeutic strategies based on its anti-tumor effects seems promising. However, the clinical use is hampered by its low biological availability. Even after the application of high doses of curcumin only low serum levels <2 μM can be gained for a view hours (Shoba et al. 1998; Anand et al. 2007) which do not show any pharmacological effect (Rashmi et al. 2005; Zhang et al. 2010). The reasons for this disadvantage can be found in its poor solubility and stability in aqueous solutions (Wang et al. 1997), and its rapid metabolism and systemic elimination (Anand et al. 2007). In the past, different strategies have been developed to enhance the biological effectiveness of curcumin. One approach is the stabilization of the molecule by complexations and conjugations (Kumar et al. 2001; Tønnesen et al. 2002; Safavy et al. 2007). Alternatively, curcumin containing nanoparticles with different strategies concerning the drug release of the compound are tested (Safavy et al. 2007; Yan et al. 2012b, 2010). Another strategy has already entered clinical trials utilizing inhibitors of curcumin metabolizing enzymes like the black pepper alkaloid piperine (Shoba et al. 1998; Singh et al. 2012). In addition numerous analogues of curcumin have been constructed and investigated with regards to stability of the molecule, bioavailability, and anti-cancer activity (Ferrari et al. 2011; Ohori et al. 2006; Ohtsu et al. 2002). When Thomas Dahl published his work about the photokilling of bacteria by curcumin (Dahl et al. 1989) a further way to enhance curcumin driven effects arose. During the following years plenty of studies appeared documenting the antimicrobial effect of curcumin combined with light (e.g. Haukvik et al. 2010, 2009; Hegge et al. 2012; Araújo et al. 2012; Dovigo et al. 2011a, b) followed by works about the anticancer effects of curcumin and light in eukaryotic cell systems. This review will sum up the present knowledge focusing on light dependent effects of low curcumin concentrations in human cells and tumor cells.

## Effect of light and curcumin on cell proliferation and viability

The treatment of HaCaT keratinocytes or primary keratinocytes with low doses (0.1–1 μg/ml = 0.27–2.7 μM) of curcumin did not show any influence on the DNA synthesis as marker for cell proliferation. Interestingly, irradiation of keratinocytes with UVA (1 J/cm²) or with visible light (5,500 lx) after treating the cells for 2 h with curcumin resulted in a clear inhibition of DNA synthesis starting already at 0.2 μg/ml curcumin indicating a strong inhibition of cell proliferation by such a combination. The chosen experimental conditions did not induce toxic membrane damage and no elevated ROS were detected. To see whether the light depending effect is cell type specific, different cell types were tested including primary skin fibroblasts, melanocytes, melanoma cells (G-361), and A-431 cells. In all cases the combination of low curcumin concentrations with light inhibited the proliferation of the tested cells (Dujic et al 2007). Similar results were gained with nasopharyngeal carcinoma cells. The cytotoxicity of curcumin on the cells was enhanced by irradiation with visible light and blue filtered light (Koon et al. 2006). Furthermore, the combination of curcumin with visible light resulted in a remarkable photocytotoxicity in HeLa cells (Banerjee et al. 2012), L929 fibroblasts (Ribeiro et al. 2012) and Lewis lung carcinoma cells (Yan et al. 2012a). In all studies described above, curcumin was combined with visible light or UVA in regard to the maximum light absorbance of curcumin at about 420 nm. Dujic et al. (2007) did not find any enhanced growth inhibiting effect if low concentrations of curcumin were combined with UVB. However, Park and Lee (2007) described a photodynamic effect of curcumin using UVB as light source in the presence of higher curcumin concentrations. From my point of view, the effects of low doses of curcumin can be amplified via light of wavelengths near the absorbance maximum of curcumin because the maximum intake of light energy is ensured under these conditions. In case of UVB and high curcumin concentrations maybe different anti-proliferative mechanisms are induced independently resulting in an additive enhancement of growth inhibition and apoptosis induction.

## Effect of light and curcumin on signal transduction pathways

It is well known that the NF-κB transcription factor can be inhibited by high concentrations of curcumin in various cell types (Singh and Aggarwal 1995; Jobin et al. 1999). Both, UVA and visible light strongly inhibit NF-κB in combination with low doses of curcumin in a concentration dependent manner (Dujic et al. 2007). In addition curcumin/UVA treatment of HaCaT cells show a clear increase of IκB-alpha indicating that low doses of curcumin combined with light suppress the survival factor NF-κB in a dual manner by suppressing the activation of NF-κB and enhancing the inhibitor of NF-κB (IκB-alpha) (Dujic et al. 2007). Furthermore, it was demonstrated that curcumin at low doses in combination with light inhibits the growth/survival kinases PKB/Akt, ERK1/2 and the related upstream receptor EGF-R (Dujic et al. 2007). The suppression of these proliferation associated pathway components can also be gained using high concentrations of curcumin without light irradiation (Squires et al. 2003; Kim et al. 2001; Chaudhary and Hruska 2003) but the combination of curcumin treatment with light enables a huge sensitization of the curcumin effects on this signal pathway.

## Effect of light and curcumin on apoptosis

One of the most interesting anti-cancer effects of curcumin is the induction of apoptosis which can be initiated with high concentrations of curcumin by both, the extrinsic and/or intrinsic way (Kim et al. 2001; Bush et al. 2001) depending from cell type and tissues. For the first time Dujic et al (2007) showed that this important anti-cancer machinery can be triggered by low curcumin concentrations if treatment is combined with UVA or visible light irradiation. Staining of nuclei by bisbenzimide revealed a marked increase of apoptotic nuclei in HaCaT cells 24 h after treatment with UVA (1 J/cm²) and 1 μg/ml curcumin. To elucidate the mechanism, the release of cytochrome c and the activation of caspases were investigated. Curcumin with light induced in HaCaT keratinocytes the release of cytochrome c from mitochondria and the activation of caspases 8, 9 and 3. Interestingly, a time-dependent dichotomy between intrinsic and extrinsic apoptosis markers could be found given that caspase 9 was activated half an hour before activation of caspase 8. Light protected controls did not show any curcumin-induced caspase activation or cytochrome c release in the tested concentration range. Similar results were described by Park and Lee (2007) who treated HaCaT cells with curcumin (5–10 μM) and subsequently irradiated cells with UVB. The authors suppose a synergistic effect of curcumin and UVB and speculate that curcumin may be clinically useful as a photosensitizer in photodynamic therapy (PDT).

## Effect of light and curcumin on tumor cells in vivo

To investigate whether the combined treatment of tumors with curcumin and visible light brings an advantage over the treatment with curcumin alone a xenograft tumor model was used. For this purpose A431 tumor cells were injected subcutaneously into the flanks of nude mice and the tumor growth was investigated in untreated control animals, in animals irradiated with visible light, in light protected but curcumin treated animals, and in animals intraperitoneally injected with curcumin and irradiated with visible light (for details s. Dujic et al. 2009). Only the combination of curcumin treatment with visible light resulted in a significant inhibition of tumor growth compared to the control groups. The average tumor volume at day 12 in curcumin/light treated mice was reduced by approximately 70 % in comparison with the untreated control group. Immunohistochemical and biochemical investigations of the tumor tissue revealed a reduction of Ki67 positive cells, a significant decrease in phosphorylation of ERK1/2 and EGF-R as well as an activation of caspase-9 in the tumors of the curcumin/light treated group compared to controls (Dujic et al. 2009). These results show clearly that combination of light and curcumin amplifies the anti-growth and pro-apoptotic effects of curcumin in a tumor model in vivo.

## Cell-specific action of curcumin

A lot of studies indicate a selective effectiveness of curcumin towards cancer cells compared with normal cells (for references s. Ravindran et al. 2009). The reason for the different sensibility to curcumin treatment is not fully understood. An enhanced cellular uptake of curcumin in tumor cells is discussed (Kunwar et al. 2008). Another consideration is that, in contrast to normal cells, most tumor cells express constitutively active NF-kB mediating their survival. The NF-kB down regulation is one of the main effects of curcumin. However, the treatment of different cell lines with curcumin combined with UVA or visible light irradiation induced growth inhibition and apoptosis induction in all tested cells including normal keratinocytes, A431 epidermoid carcinoma cells, HaCaT-keratinocytes, normal fibroblasts, normal melanocytes and different melanoma cell lines (Dujic et al. 2007, and unpublished results). Further investigations are necessary to elicit if also this curcumin based therapy concept may operate selectively.

## Molecular action of curcumin

**Fig. 1:**
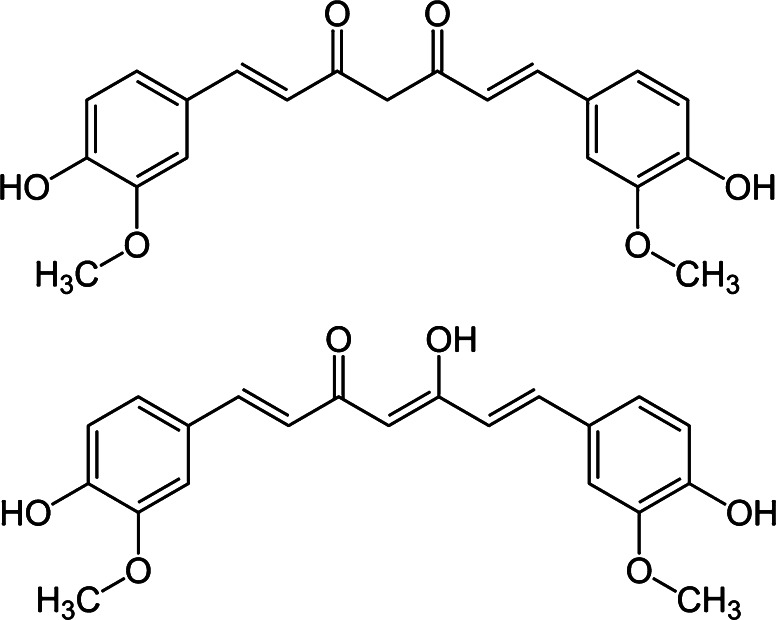
Chemical structure of curcumin

The chemical structure of curcumin, *bis*(4-hydroxy-3-methoxyphenyl)-1,6-diene-3,5-dione (Fig. 1), was elucidated by Lampe and Milobedzka (1913) and further described in detail under different physical conditions by Roughley and Whiting (1973). The molecule contains functional parts like phenoxy groups, methoxy groups and the tautomerism between enol- and keto-structures. The energetically more stable enol-form is stabilized by intramolecular hydrogen boundings which may be partly responsible for its biological effects. The double bindings of the aliphatic chain between the two phenol rings are important for the mode of action of curcumin. After elimination of the double bonds the apoptosis inducing activity of curcumin was lost (Pae et al. 2007). The anti-oxidative and the radical scavenger properties of curcumin are thought to be supported by the phenoxy groups (Araújo and Leon 2001). Other authors show that curcumin can also act as a radical donor. High concentrations of curcumin enhance the intracellular concentration of ROS subsequently causing the induction of cell cycle arrest and apoptosis (Chen et al. 2010; Weir et al. 2007). The radical transmitting effect of curcumin can be enhanced by light leading to an effective photochemical strategy against microorganisms (Araújo et al. 2012, Dahl et al. 1994). However, at low concentrations the radical scavenging property of curcumin seems to be predominant (Premanand et al. 2006) even in combination with light (Dujic et al. 2007). No significant induction of ROS was detected by treatment of cells with low doses of curcumin alone or in combination with 1 J/cm² UVA. On the contrary, when ROS generation was induced with high doses UVA (20 J/cm²) curcumin reduced UVA induced ROS in a concentration dependent manner using 0.2–1 μg/ml curcumin (Dujic et al. 2007). Therefore, I suppose that low doses of curcumin combined with 1 J/cm² UVA or visible light induce apoptosis by other mechanisms than by ROS generation. In addition, the impact of different curcumin concentrations on cell membrane structure was demonstrated using a combination of solid-state NMR and different scanning calorimetry techniques. It was shown that curcumin disturbs the membrane fluidity and changes the conformation and function of integral membrane proteins dependent from the concentration used (Barry et al. 2009; Ingolfsson et al. 2007). Despite intensive efforts to elucidate the exact chemical mode of action and the role of light in the enhancing effect of curcumin, a major molecular mechanism could not be identified so far and further investigations are indispensable. Interestingly, photo-bleaching of curcumin resulted in a total loss of its light dependent anti-tumor activities (Dujic 2008). Curcumin binds directly to a lot of different proteins including signaling molecules, enzymes and carrier proteins (reviewed in Gupta et al 2011). The close proximity in curcumin-protein complexes makes light driven interactions most likely. Barik et al. (2003) investigated the binding of curcumin on proteins using BSA. They showed a high affinity of curcumin to BSA accompanied by a strong enhancement of fluorescence emission and a blue-shift of the fluorescence spectrum of curcumin. Considering these findings a light dependent energy transfer via curcumin may enhance the influence of curcumin on protein functions resulting in the described effects on cell physiology. However, this hypothesis needs further investigations.

## Conclusions and perspectives

**Fig. 2:**
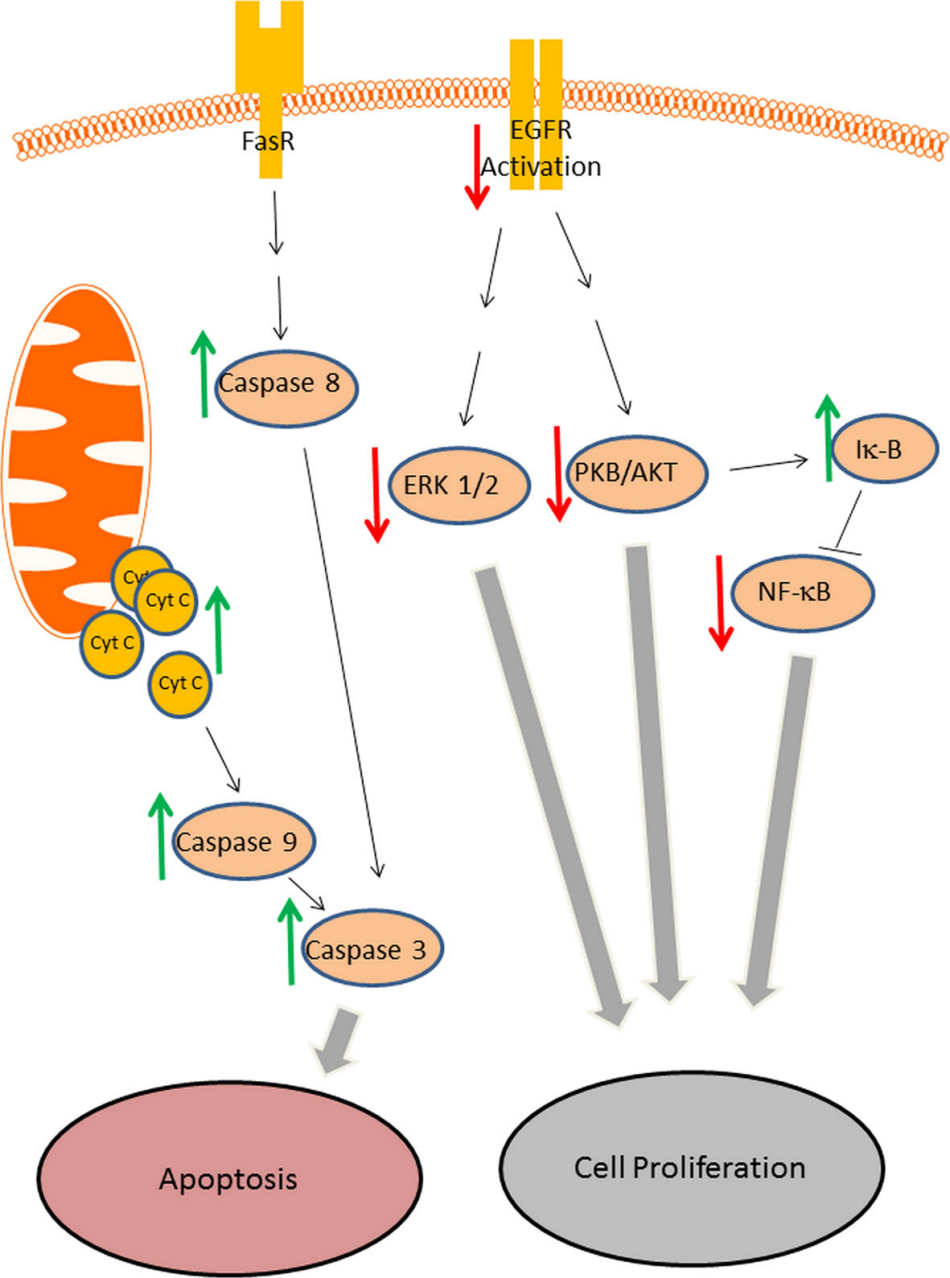
Schematic diagram of curcumin/light affected pathways. *Green arrows* enhancement, *red arrows* inhibition

In vitro- and in vivo-experiments showed that very low concentration of curcumin is sufficient to induce anti-tumor effects if the treatment is combined with UVA or visible light irradiation. The used doses of visible light or UVA alone did not show any significant anti-tumor effects. Just the combination of light with curcumin induced the described anti-tumor activities. The influence of low curcumin concentrations combined with light on key factors of growth and apoptosis was shown in vivo and in vitro (Fig. 2). Consequently, the difficulty of the low bioavailability of curcumin can be compensated by a combination of curcumin with light treatment. The pre-clinic experiments argue for starting the development of clinical therapy concepts based on curcumin and light. Further investigations are necessary to elucidate the physico-chemical mechanism of light dependent curcumin effects.
